# Nurse practitioner and midwife antibiotic prescribing in Australia

**DOI:** 10.18332/ejm/162439

**Published:** 2023-05-26

**Authors:** Glenda Hawley, Aaron Grogan, Treasure McGuire, Mieke van Driel, Samantha Hollingworth

**Affiliations:** 1 School of Nursing, Midwifery and Social Work, The University of Queensland, St Lucia, Australia; 2 School of Pharmacy, The University of Queensland, Woolloongabba, Australia; 3 Mater Pharmacy Services, Mater Health Services, South Brisbane, Australia; 4 Faculty of Health Sciences and Medicine, Bond University, Robina, Australia; 5 Primary Care Clinical Unit, The University of Queensland, Herston, Australia; 6 General Practice Clinical Unit, Faculty of Medicine, University of Queensland, Brisbane, Australia

**Keywords:** nurse practitioners, midwives, antimicrobial resistance, antibacterial agents, prescribing, non-medical

## Abstract

**INTRODUCTION:**

Antimicrobial resistance is of global significance. To reduce the risk of harm associated with antibiotic prescribing in Australia, a recent strategy to tackle antimicrobial resistance has included non-medical prescribers. Traditionally, antibiotic prescribing has been the domain of the medical profession but, more recently, nurse practitioners and endorsed midwives have been authorized to prescribe antibiotics. This study describes the antibiotic prescribing practices by nurse practitioners and endorsed midwives in Australia, with clinical implications for international settings.

**METHODS:**

This was a retrospective analysis of routinely collected aggregated data of anonymous individuals. Data on dispensed prescriptions of antibiotics were obtained from the Australian Department of Human Services, for the period 2005–2016. All antibiotics were allocated to a spectrum class (narrow, moderate, broad). Analysis using descriptive statistics was undertaken to determine the antibiotic prescribing patterns of nurse practitioners and endorsed midwives.

**RESULTS:**

Nurse practitioners have been prescribing within Australia since 2000, and midwives since 2012. Nurse practitioner antibiotic written scripts increased from 3143 during 2005–2011 to 34615 in 2012–2016, while antibiotic written scripts by midwives increased from 2012 (n=2) to 2016 (n=469). Nurse practitioners and midwives prescribed similar classes of antibiotics. These professionals are important non-medical prescribers and are increasingly writing antibiotic prescriptions.

Both nursing and midwifery cohorts complete accredited education programs, albeit with some differences in structure.

**CONCLUSIONS:**

When prescribing antibiotics, nurse practitioners and midwives are following evidenced-based therapeutic guidelines. They are increasingly relevant clinicians prescribing antibiotics, particularly in acute and primary care settings, which has relevance in global antimicrobial strategies.

## INTRODUCTION

Antibiotics extend human life expectancy by up to 20 years^1^. More particularly, antibiotics have reduced the rates of infections causing meningitis, respiratory infections and childbirth-related sepsis^2^. Specifically, in midwifery, childbirth-related deaths have decreased from 3 in 100 in the 1920s to 3 in 100000 in recent years^2^. Despite these gains, antimicrobial resistance is predicted to be responsible for more deaths than cancer by the year 2050^3^. Alarmingly, Australia is one of the highest prescribers of antibiotics in the world^4^. In response, the Australian Government developed a national antimicrobial resistance strategy in 2015 to address the threat from antibiotic misuse and resistance^5^. The strategy aims to provide antimicrobial stewardship (AMS) to all sectors of health care, whereas most AMS strategies have been undertaken in acute hospital settings by medical professionals rather than in primary or community healthcare settings^4^.

As non-medical health professionals, nurses and midwives play an important role in the AMS strategy, as practicing prescribers, both worldwide and in Australia^5,6^.

The Australian Health Practitioners Regulation Authority (AHPRA) describes the requirements to attain professional endorsement as nurse practitioner (NP) and endorsed midwife. Subsequently NPs and endorsed midwives are allowed to access and prescribe within the Pharmaceutical Benefits Scheme in line with medical prescribers^7^.

The legislated NP role was introduced in Australia in 2000, with nurse practitioners authorized to prescribe. This role was envisaged to reduce inequities in healthcare and provide accessible and efficient care to marginalized groups whose healthcare needs were largely unmet^8^. The NP operates within a defined scope of practice which is regulated and endorsed at a local health service level. This scope of practice fits within one or more meta-specialties, each of which is defined by specific educational and practice standards. The meta-specialities include primary health, emergency and acute care, ageing and palliative care, mental health, chronic and complex care, and child and family care^9^.

Midwives in Australia were given prescribing rights in 2010 following a review of national maternity services. In 2015, Small et al.^10^ reported that only 59% of the Medicare-eligible midwives were endorsed to prescribe medications. This low uptake was attributed to the lack of an accredited Graduate Certificate program and, subsequently, to differing legislation among the States and Territories^10^. In the most recent Nursing and Midwifery Board of Australia (NMBA) registrant data, all eligible midwives now are endorsed to prescribe^11^. Midwives can prescribe antibiotics in the antenatal, intrapartum, and postnatal stages of pregnancy, and this is integral to improving the continuous model of care with the woman by ensuring timely treatment, effective monitoring of complications, and comprehensive education^10,12^.

Following an undergraduate 3-year degree, nurses and midwives complete further education to qualify as eligible prescribers of medications, including antibiotics. Nurse practitioners need to complete an endorsed Master’s program and midwives a specific prescribing qualification (Graduate Certificate) through an accredited tertiary educational facility, while also adhering to relevant state and territory legislations^13,14^.

The AMS strategy to incorporate NPs and midwives in the prescribing review is particularly relevant as these professions play key roles within the contemporary healthcare environment, encompassing the diversity of acute and primary healthcare settings^15,16^. In Australia (December 2022), there were 2494 endorsed NPs (only 123 in 2012) and 968 endorsed midwives (only 1 in 2012)^11^. These NPs and midwives work in a range of practice settings from acute to primary healthcare, in urban and rural environments^12^. Therefore, it is reasonable to consider the significant and developing role that NPs and midwives play in embedding AMS principles in their practice^17^.

Prescribing decision-making can be complex and challenging, with several influences identified among medical prescribers such as lack of knowledge, familiarity, or failure to comply with the AMS strategy policy^17^. There are few studies describing the prescribing patterns of and influences on non-medical prescribers^18^. Hence, this study reports on the antibiotic prescribing practice records of NPs and endorsed midwives in Australia.

## METHODS

This was a retrospective, observational study using data on dispensed prescriptions of antibiotics subsidized on the Pharmaceutical Benefits Scheme (PBS). These data were obtained from the Australian Department of Human Services. Because individuals were anonymous, no ethical approval and informed consent were necessary. We conducted and reported this research in accordance with the guideline for reporting of studies conducted using observational routinely collected health data (RECORD)^19^ and extended the checklist to include reporting guidelines specific to pharmacoepidemiological research (RECORD-PE)^20^. The data describes PBS only prescribing by NPs and endorsed midwives, according to: 1) antibiotic; 2) spectrum; and 3) geographical area (metropolitan, regional, rural); between 2005 and 2016 by quarter^6^.

All antibiotics were allocated to a spectrum class (narrow, moderate, broad), e.g. amoxicillin + clavulanic acid and doxycycline were classified as broad spectrum, while amoxicillin and cefalexin were classified as moderate spectrum^21,22^. We analyzed the data using descriptive statistics (Stata Statistical Software: Release 14. College Station, TX: StataCorp LP).

## RESULTS

### Antibiotic prescribing by NPs and endorsed midwives

The antibiotics prescribed by NPs and midwives differ. Antibiotic prescribing by NPs increased from 3143 prescriptions in the period 2005–2011 to 34615 in 2016. To note, midwives only issued two prescriptions in 2012, but steadily increased over the next 5 years to 469 in 2016 (Table 1). In 2016, NPs represented 3.75% of all non-medical prescribers, and midwives only 0.05%. Non-medical prescribers represented 3.5% of all (medical plus non-medical) prescribers in 2016 (Table 1).

**Table 1:** Antibiotic dispensed use by non-medical and medical health professionals for the period 2005–2011 and from 2012 to 2016

*Health professionals*	*2005–2011*	*2012*	*2013*	*2014*	*2015*	*2016*
**Non-medical**						
Nurse practitioner	3143	12889	17320	25149	31123	34615
Midwife	0	2	66	247	304	469
Other	2478631	809583	948595	925623	910769	887292
Total	2481774	822474	965981	951019	942196	922376
**Medical**						
Total	85341238	23443042	26279246	27188046	27484538	26490599

### Antibiotic prescribing by spectrum and medicine

NPs prescribed both broad- and moderate-spectrum antibiotics in roughly equal numbers until 2015, and there was a change to preferential prescribing of broad spectrum (49%) versus moderate spectrum (39%) in 2016. Only 15% of prescriptions were for narrow-spectrum antibiotics. For midwives, about 45% were moderate-spectrum and about 37% were narrow-spectrum antibiotics on average between 2014 and 2016 (Table 2).

**Table 2:** Antibiotic dispensed use by spectrum of antibiotic for nurse practitioners and midwives, from 2012 to 2016

*Spectrum*	*2012 n (col %)*	*2013 n (col %)*	*2014 n (col %)*	*2015 n (col %)*	*2016 n (col %)*	*Total n (col %)*
**Nurse practitioners**						
Broad	5507	7544	10969	14343	16219	33920
	42.7	43.6	43.6	46.1	46.9	45.0
Moderate	5438	7210	10414	12361	13449	50214
	42.2	41.6	41.4	39.7	38.9	40.4
Narrow	1944	2566	3766	4419	4947	18106
	15.1	14.8	15	14.2	14.3	14.6
**Midwives**						
Broad	1	3	38	57	88	187
	50.0	5.0	15.0	19.0	19.0	17.0
Moderate	1	24	114	133	221	492
	50.0	36.0	46.0	44.0	47.0	45.0
Narrow	0	39	95	114	160	409
	0	59.0	39.0	37.0	34.0	38.0

Of interest is that prescribing of the most widely used antibiotics was similar for both professions. The preferred antibiotic was cefalexin (moderate) followed by amoxicillin with clavulanic acid (broad) and amoxicillin (moderate) (Table 3). There were some differences between the professions. Regarding broad-spectrum options, NPs prescribed doxycycline, roxithromycin, and trimethoprim, whereas midwives mostly prescribed azithromycin and nitrofurantoin (Table 3).

**Table 3:** Antibiotic dispensed use by individual medicine and spectrum, prescribed by nurse practitioners and midwives

*Spectrum*	*Antibiotics prescribed*	
	*Nurse practitioners (rank)*	*Midwives (rank)*
**Same prescribing**		
Moderate	Cefalexin (1)	Cefalexin (1)
	Amoxicillin (3)	Amoxicillin (3)
Broad	Amoxicillin with clavulanic acid (2)	Amoxicillin with clavulanic acid (2)
Narrow	Flucloxacillin (no rank)	Flucloxacillin (no rank)
**Different prescribing**		
Broad	Trimethoprim	Nitrofurantoin
	Doxycycline	Azithromycin
	Roxithromycin	
Narrow	Phenoxymethylpenicillin	Benzylpenicillin
		Clindamycin

### Antibiotic prescribing by spectrum and geographical area

The prescribing of antibiotics by NPs and midwives by spectrum and geographical area was also examined. NPs predominantly prescribed broad-spectrum antibiotics in major cities (49%) and remote areas (48%). In contrast, midwives prescribed narrow-spectrum antibiotics in major cities (40%) and vastly more moderate-spectrum antibiotics in remote areas (85.9%).

## DISCUSSION

This study describes the prescribing of antibiotics by Australian NPs and midwives. NPs prescribe many more antibiotics than midwives, and this reflects the large differences in the respective cohorts. NPs work in many clinical specialties, so the available cohort of patients is much greater. The incremental increase in total prescriptions likely reflects the increased number of NPs and midwives qualified to prescribe^17^. Additionally, the variation in prescriber numbers between NPs and endorsed midwives can be attributed to more accredited NP programs in comparison to endorsed midwife programs^7^. Fong et al.^23^ state that 78% of NPs consider prescribing as part of their professional role, despite the fact that all NPs complete prescribing education as part of their professional qualification. In addition, with high numbers of NPs working within acute and primary care specialties, the opportunity for a need to prescribe antibiotics is much more frequent. This is in comparison to endorsed midwives who work exclusively within a specific woman-centered model of care facility^10^.

Highlighted was that the type and spectrum of antibiotic therapy prescribed varies across NPs and midwives. The reasons remain to be elucidated but may reflect educational content or influences within clinical practice. Professional confidence is a major factor in prescribing practices among NPs, with a known reluctance to change or amend current medication therapies prescribed by others^24^. NP students, compared with dental and medical students, are less confident about prescribing despite similar educational preparedness within prescribing frameworks^25^. These two factors may lead to prescribing similarities based not on clinical intuition but reluctance to deviate from common prescribing patterns.

It is important to understand that both nurse practitioners and midwives are required by law to complete education with an accredited program prior to legally prescribing medicines, including antibiotics. Consequently, there is a difference in the type of qualification and facility that are accredited to provide the necessary training for both midwives and nurse practitioners. Nurse practitioners are endorsed to practice after the completion of both an accredited Master’s program and the supply of evidence supporting 5000 hours of advanced clinical practice. Midwives can apply for endorsement after completion of an NMBA approved program of study at post-graduate level and also demonstrate evidence of the equivalence of three years’ full-time clinical practice, across the maternity care spectrum^7,26^. Currently in Australia, there are 11 accredited Master of Nurse Practitioner programs, and five graduate medication certificate courses available for midwives^7,26^.

With regard to which antibiotics are issued, prescribers are influenced by varying factors that include patient expectations, diagnostic ambiguity, other healthcare professionals, and national guidelines^27^. While our study examined national data, Courtenay et al.^27^ surveyed prescribers who enumerated the type and volume of antibiotics prescribed by NPs. Buckley et al.^28^ reported that NP prescribing patterns were similar to non-NPs when reviewing adherence to antibiotic prescribing guidelines. Half of Australian NPs used the Australian Medicines Handbook, and over 30% used the electronic therapeutic guidelines to determine appropriate therapy^28^. The antibiotics prescribed also correlated with recommended practice in the general practice therapeutic guidelines^25^. For example, benzylpenicillin is prescribed by midwives, most likely for group B streptococcal infections in intrapartum care and endorsed in the guidelines^27,29^.

In primary healthcare settings, NPs and midwives follow a continuity-of-care model and hence spend more time with patients and women, which leads to a more holistic understanding of the individual’s ongoing healthcare needs and social circumstances. Although this could imply that NPs or midwives might be persuaded to prescribe more freely, Ness et al.^15^ consider the continuity-of-care model to be an important step to favorably contribute to AMS. Nurses can help in managing health issues and influencing behavior to use non-antibiotic approaches^15^. They have an important role and a duty of care in assessing patient need and prescribing in the primary and acute healthcare sectors. This is done using an evidence-based approach, incorporating and addressing AMS strategies^30^. Nurses also have a key role in preparing and administering antibiotics, and monitoring side effects^30^.

While there are population differences between Australia and the rest of the globe, the findings from this study can be used to illuminate and promote the possible prescribing practices of local nurses and midwives.

### Strengths and limitations

This study is the first to describe antibiotics prescribing by NPs and midwives in Australia over a decade, and we highlight that they appear to be following antimicrobial stewardship and evidenced-based therapeutic guidelines. However, there are two main limitations. Firstly, we do not know the indications for prescribing particular antibiotics. Secondly, not every NP or midwife will prescribe antibiotics. For example, while 78% of NPs prescribe antibiotics as part of usual practice, a mental health NP will primarily prescribe psychotropic medicines and will not have the same expertise in antibiotic prescribing as an acute or primary-care trained NP^31^. Midwives could be expected to prescribe antibiotics in both the antenatal and postnatal periods. Exploring indications for antibiotic prescription by nurse practitioners and midwives, along with antibiotic prescribing curricula in their training programs, will be instructive.

### Implications for research, policy and practice

The factors influencing prescribing by medical practitioners are well researched, but we know much less about NPs and midwives. Research suggests that NPs were more confident in educating patients and monitoring the effects of pharmacological interventions but were less confident in adjusting or ceasing medicines initiated by other prescribers^24^. The initial prescribing confidence of NPs could be attributed to the advanced experience that NPs have at registered-nurse level, where educating patients and monitoring interventions is a cornerstone of contemporary practice, and we would expect the same for midwives. The reluctance to adjust or cease medicines prescribed by others is likely a remnant of the education of NP and midwife prescribers and an inconsistency in the collaborative relationships within which they are bound to or feel obliged to operate in^32^.

It would be valuable to explore antibiotic prescribing curricula in the respective training programs for NPs and midwives. The current training programs are endorsed by the Australian Nursing and Midwifery Accreditation Council, but it would be interesting to compare the AMS educational activities for medical students, including diagnostic ambiguity and simulations^33^. It would be instructive to interview NPs and midwives about the processes and resources used to diagnose and prescribe antibiotics. Combining antimicrobial stewardship education for nurse practitioners, midwives, and medical students will promote consistent prescribing practices and may strengthen compliance with strategy policy.

An approach described by Paterson et al.^34^ is to maintain reflective logs, as a novel tool to demonstrate prescribing competence linked directly to clinical experience and validating AMS principles^34^. The logs could be leveraged to determine the factors that influence when and what classes of antibiotics are prescribed by NPs and midwives. Combining medical and non-medical practitioners in educational opportunities in AMS would promote consistent approaches to prescribing practices. Integrating strategies in training programs is beneficial in improving standardized approaches, along with guidelines to managing healthcare issues requiring antibiotics^31^. Activities such as ‘Antibiotic Awareness Week’ could provide a focus for AMS education for all prescribers^17^.

## CONCLUSIONS

We have described the number, spectrum, and type of antibiotics prescribed by NPs and midwives in Australia within the context of an Australian AMS strategy. NPs and midwives are increasingly important as clinicians prescribing antibiotics, particularly in acute and primary care settings. Although unable to be confirmed, the findings from the data appear to demonstrate that NPs and midwives follow therapeutic guidelines. We do, however, need to better understand the details such as discipline subgroups and indications for prescribing. An audit of training curricula and revisions as required will ensure robust diagnostic skills contributing to appropriate use of antibiotics and reduced antimicrobial resistance. While this study highlights a small sample of nursing and midwifery prescribing practices, the findings are useful to apply internationally.

## Data Availability

The data supporting this research are available from the authors on reasonable request.
